# Relationship between frequency of yogurt consumption at 1 year of age and development at 3 years of age: The Japan Environment and Children’s Study

**DOI:** 10.1371/journal.pone.0308703

**Published:** 2024-12-04

**Authors:** Hiroko Hirai, Tomomi Tanaka, Kenta Matsumura, Akiko Tsuchida, Yuichi Adachi, Chihaya Imai, Hidekuni Inadera, and the Japan Environment and Children’s Study Group

**Affiliations:** 1 Department of Pediatrics, Faculty of Medicine, University of Toyama, Toyama, Japan; 2 Toyama Regional Center for JECS, University of Toyama, Toyama, Japan; 3 Department of Public Health, Faculty of Medicine, University of Toyama, Toyama, Japan; 4 Pediatric Allergy Center, Toyama Red Cross Hospital, Toyama, Japan; Universidade dos Açores Departamento de Biologia: Universidade dos Acores Departamento de Biologia, PORTUGAL

## Abstract

**Background:**

Multiple observational studies have demonstrated the health benefits of yogurt, which are considered due to yogurt’s positive effects on the gut microbiota. It is also known that the intestinal microbiota is associated with cognitive and emotional functions. Although the intake of probiotics has been reported to improve neurodevelopmental symptoms in children, no large-scale studies have examined the association of yogurt consumption in early childhood with later neurodevelopmental status. In this study, we examined the association between frequency of such consumption at 1 year of age and the children’s subsequent neurodevelopmental status.

**Methods:**

We studied children’s dietary consumption of yogurt at age 1 year and their neurodevelopment at age 3 years from data gathered from70,276 mother–child pairs enrolled in the Japan Environment and Children’s Study. We collected data from pregnant women whose consent was obtained after the study was explained to them at participating facilities in the target area. Frequency of yogurt consumption was categorized into 0, 1–2, 3–4, and ≥ 5 times/week based on a questionnaire about the child’s diet completed by the mother. Developmental delay was assessed using the Ages and Stages Questionnaires, Third Edition (ASQ-3™) in five domains: communication, gross motor, fine motor, problem solving, and personal-social. Using the results of the group that did not consume yogurt as a reference, multivariate logistic regression analysis was performed to compare the neurodevelopment of children according to frequency of yogurt consumption. For the covariates, items related to the socio-economic background and children’s neurodevelopment were selected with reference to previous studies.

**Results:**

Consumption of yogurt 1–4 times/week was associated with a reduced risk of developmental delay in all ASQ-3 categories(adjusted odds ratios, 0.71–0.87). However, the risk of developmental delay was not necessarily reduced with yogurt consumption ≥5 times/week (adjusted odds ratios, 0.84–0.96).

**Conclusion:**

Yogurt consumption habits at 1 year of age were associated with a lower risk of developmental delay at 3 years of age. However, the association was less apparent when yogurt was consumed more frequently.

Possible mechanisms by which yogurt intake affects neurodevelopment include neurotransmitters produced by intestinal bacteria as well as the suppression of intestinal inflammation through improvements in the intestinal environment. Regular intake of yogurt in early childhood may have a positive association with neurodevelopment, but it is hoped that clearer links will be found in the future through intervention studies.

## Introduction

Yogurt is a nutrient-rich food that is available worldwide, and multiple observational studies have suggested its health benefits. In adults, the consumption of fermented foods such as yogurt has been reported to be associated with reduced obesity and lower risks of type 2 diabetes, metabolic syndrome, and heart disease [1]. In children, yogurt consumption has been shown to improve symptoms and prevent the onset of acute diarrhea and atopic dermatitis [1, 2]. These beneficial effects of yogurt consumption are thought to be mediated by mechanisms such as improvement of the intestinal microbiota and promotion of intestinal immunity.

Recent reports suggest that the gut microbiota influences cognitive and emotional functions from the perspective of gut–brain interactions. In a clinical trial using a mouse model of Alzheimer’s disease, yogurt consumption alleviated symptoms by modulating the gut microbiota and resolving dysbiosis, a condition that can negatively affect brain function and behavior [3]. In studies in children, probiotic consumption was associated with symptom improvement in autistic spectrum disorder (ASD) [4–6].and regular yogurt consumption tended to be associated with a lower rate of sleep deprivation in a general pediatric population [4]. Sleep disorders are frequently associated with neurodevelopmental disorders, suggesting that there is some association between yogurt consumption habits and neurodevelopment [5, 6]The mechanism by which the gut microbiota influences neurodevelopment is thought to involve the action of neurotransmitters such as GABA in favor of certain gut bacteria as well as the inhibition of inflammation in the gut [10–14].

Because yogurt is a familiar food containing probiotics and can be consumed from infancy, it may have the potential to influence the composition of the intestinal microbiota in infancy as well as later in life. Given the association of the gut microbiota with cognitive function and emotional motility, we hypothesized that yogurt consumption habits in early childhood would be associated with later development. To our knowledge, no large-scale studies have examined the association of yogurt consumption in early childhood with neurodevelopment, and therefore in this study we examined whether the consumption frequency at 1 year of age was associated with neurodevelopmental status at 3 years of age [6, 15]

## Materials and methods

### Study population

This study analyzed data obtained in the Japan Environment and Children’s Study (JECS), a nationwide, government-led birth cohort study investigating the relationship of environmental factors with child health and development. The detailed design and baseline characteristics of the Japan Environment and Children’s Study (JECS) have been reported elsewhere [7].

The total number of pregnancies resulting in delivery was 100,778, of which 51,402 (51.0%) involved program participation by male partners. Excluding multiple registrations of pregnancies by the same woman, the study included 95,248 unique mothers and 49,189 unique fathers. The 100,778 pregnancies involved a total of 101,779 fetuses and resulted in 100,148 live births. Although the coverage of children by JECS in 2013 (the number of live births registered in JECS divided by the number of all live births within the study areas) was around 45%, the data on the characteristics of the mothers and children we studied showed marked similarity to those obtained from Japan’s 2013 Vital Statistics Survey. The distribution of baseline profiles did not differ significantly between the full population of approximately 100,000 mothers and the sub-population of approximately 50,000 mothers with male partners who participated in the study. The data on mother and child characteristics from the JECS are similar to those from the Japanese Vital Statistics and are considered to be representative of the population.

**Fig 1:**
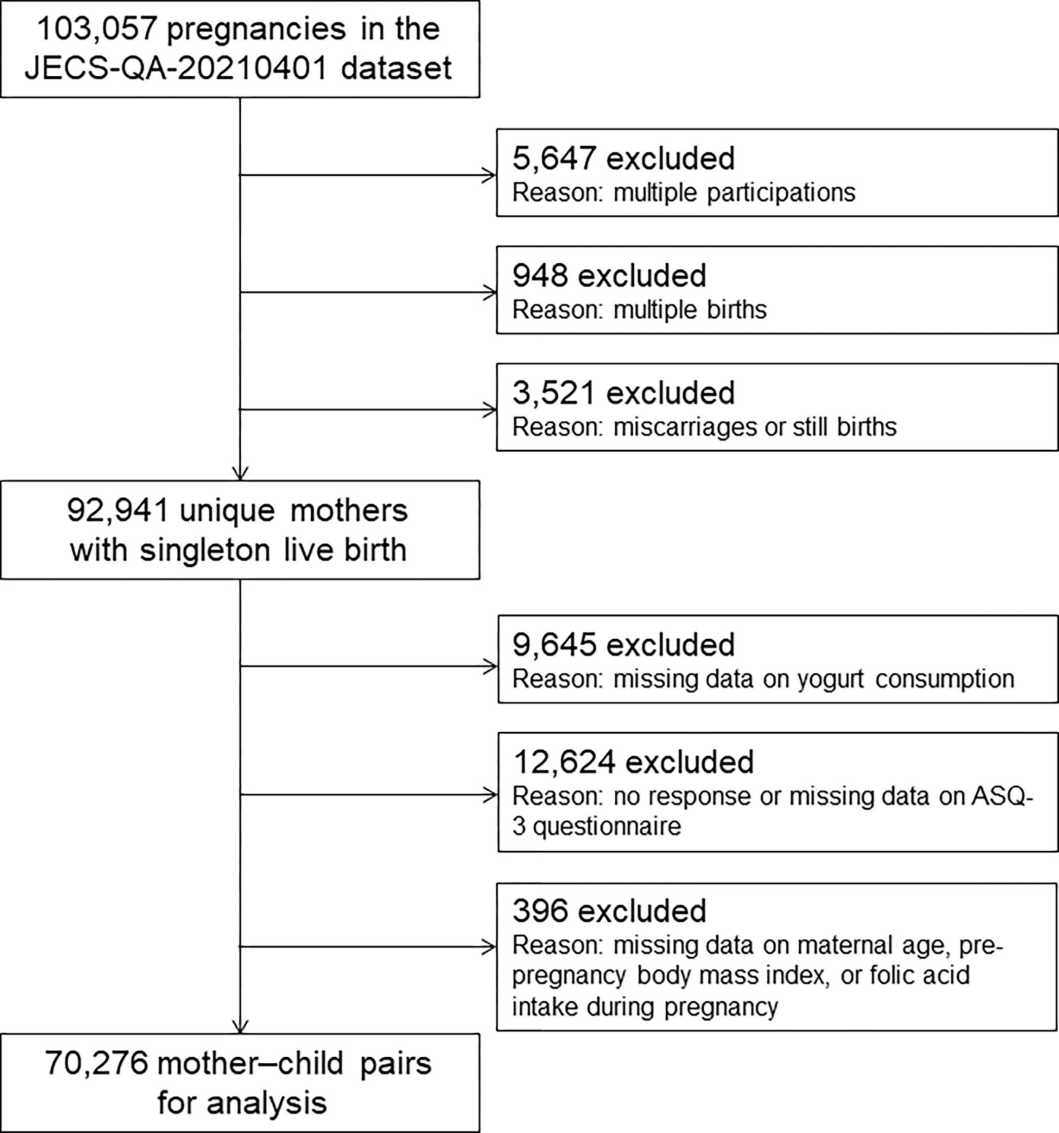
Flow diagram of the participant selection. This study involved 103,057 pregnant women; 5,647 were excluded because of multiple enrollments, 948 because of multiple births, and 3,521 because of miscarriages or stillbirths. Of the children born to the remaining 92,941 mothers, 9,645 were excluded because their yogurt consumption status was unknown, 12,624 due to no response or completely missing data on ASQ-3, and 396 due to missing data on maternal age, pre-pregnancy BMI, or folic acid intake, resulting in a final sample of 70,276 mother–child pairs.

The children participating in JECS were selected from 15 regions across the country during the period January 2011 to March 2014 [7–9]. The present study analyzed the jecs-qa-20210401 dataset released in April 2021. From a total of 103,057 pregnancies, 5,647 were excluded because of multiple enrollments, 948 because of multiple births, and 3,521 because of miscarriages or stillbirths. Of the children born to the remaining 92,941 mothers, 9,645 were excluded due to unknown yogurt consumption status, 12,624 due to no response or completely missing data on the ASQ-3, and 396 due to missing data on maternal age, pre-pregnancy BMI, or folic acid intake, resulting in a final sample of 70,276 mother–child pairs (Fig 1). All data were obtained from a questionnaire completed by mothers and/or caregivers on their child’s yogurt consumption at age 1 year.

The authors assert that all procedures contributing to this work comply with the ethical standards of the relevant national and institutional committees on research involving human participants and with the Helsinki Declaration of 1975, as revised in 2008and its later amendments. The JECS protocol was reviewed and approved by the Ministry of the Environment’s Institutional Review Board on Epidemiological Studies (Approval No. 100910001) and the ethics committees of all participating institutions. Written informed consent was obtained from all participants in JECS. The present study was also approved by the Ethics Committee of the University of Toyama (R2018032).

### Survey of frequency of yogurt consumption

To assess the frequency of probiotic intake via the consumption of yogurt, the following question was included in the self-administered questionnaire that mothers and/or caregivers completed 1 year after delivery: “How many times a week does your child eat yogurt?” The response options were 0 times/week, 1–2 times/week, 3–4 times/week, 5–6 times/week, 1 time/day, 2 times/day, and 3 or more times/day. The questionnaires were returned by post by a stipulated deadline. Referring to previous reports on the frequency of yogurt consumption and its health effects, we categorized the participants by consumption frequency as follows: none, 1–2 times/week, 3–4 times/week, and ≥ 5times/week [4].

### Neurodevelopment

Children’s neurodevelopment at age 3 was assessed using the Ages and Stages Questionnaires, Third Edition(ASQ-3™), an age-specific, structured, parent-completed, child monitoring system. The ASQ-3 consists of a set of well-validated questionnaires and has been recommended by the United Nations Children’s Fund to verify whether children have typical neurological development. The Japanese version of the ASQ-3 has been validated [10] and has been used in several previous studies [11, 12]. The ASQ-3 assesses development in the following five domains: (a) communication: language skills, such as babbling, vocalizing, listening, and understanding; (b) gross motor: arm, body, and leg movements during movement and play; (c) fine motor: hand and finger movements; (d) problem solving: problem-solving skills, learning, and playing with toys; and (e) personal-social: self-help skills, solitary social play, and play with toys and others. Screen-positive cases for each domain are defined as those with scores at or below the cutoff values [10]. Taking early delivery into account, if a parent’s completion dates at 1.5,2.0,2.5, and 3.0 years were not within±1 month of the estimated delivery date, the data were treated as missing values in accordance with the scoring guidelines.

### Covariates

We used the following variables as potential confounders for mothers: age during pregnancy (< 25 years, 25–30 years, 30–35 years, and > 35 years); folic acid intake during pregnancy from the Food Frequency Questionnaire [13] (≤ 165 μg, 166–228 μg, 229–312 μg, ≥ 313 μg); physical activity during pregnancy from the International Physical Activity Questionnaire [14, 15] (≤ 0.152 Metabolic equivalents [Mets]∙h/week, 0.157–1.177 METs∙h/week, 1.178–3.305 METs∙h/week, ≥ 3.305 METs∙h/week); BMI (< 18.5 kg/m^2^, 18.5–25 kg/m^2^,≥ 25 kg/m^2^);parity (primipara, multipara); smoking status(never, former, current); alcohol consumption status (former [stopped before pregnancy],former [stopped upon learning of pregnancy], current); marital status 6 months after birth (married, single); highest educational level (junior high school or high school, vocational school or junior college, university or graduate school); highest educational level of the mother’s partner (junior high school or high school, vocational school or junior college, university or graduate school); employment status 1 year after birth (not employed, employed); annual household income (< 4 million yen, 4–6 million yen, ≥ 6 million yen);and use of antibacterial medication history during pregnancy (no, yes).

As potential confounders for children, we considered gestational week(< 37 week, ≥ 37 week), sex (boy, girl), cesarean delivery (yes, no), congenital anomaly (yes, no), and being breastfed at age 1 year (no, yes).These covariates include standard variables for socioeconomic status and living environment. In addition, we selected several variables in terms of the possibility of impact on exposure and/or outcome. The categorization of these variables was performed according to usual medical practice or common practice in Japan and/or with reference to our previous studies [11, 12, 16, 17].

### Statistical analysis

To estimate the risk of neurodevelopmental delay according to the frequency of yogurt consumption, the children were divided according to quartiles for consumption (none, 1–2 times/week, 3–4 times/week, and ≥ 5 times/week), and odds ratios and 95% confidence intervals were determined by multivariate logistic regression analysis. All statistical analyses were performed using SAS ver. 9.4(SAS Institute Inc.,Cary, NC). Missing covariate data were included as categorical variables in the adjusted model.

## Results

Table 1 shows participant characteristics according to quartiles for yogurt consumption at age 1 year. Associations were found between consumption frequency and age during pregnancy, physical activity during pregnancy, household income, highest educational level of the mother’s partner, employment status, alcohol consumption status, smoking status, parity, and being breastfed at age 1 year.

**Table 1:** Participant characteristics according to quartile for yogurt consumption at age 1 year.

Variable		Frequency of yogurt consumption at age 1 year (times/week)						
	0(none)		1–2		3–4		≥ 5	
	N	(%)	N	(%)	N	(%)	N	(%)
Subtotal	14,423	(20.5)	21,953	(31.2)	15,524	(22.1)	18,376	(26.2)
**Age during pregnancy, year**								
< 25	1,295	(9.0)	2,153	(9.8)	1,299	(8.4)	1,322	(7.2)
25–< 30	3,875	(26.9)	6,361	(29.0)	4,445	(28.6)	4,966	(27.0)
30–< 35	5,355	(37.1)	7,751	(35.3)	5,643	(36.4)	6,620	(36.0)
≥ 35	3,898	(27.0)	5,688	(25.9)	4,137	(26.6)	5,468	(29.8)
**Pre‐pregnancy body mass index, kg/m2**								
< 18.5	2,323	(16.1)	3,499	(15.9)	2,549	(16.4)	3,081	(16.8)
18.5–< 25	10,755	(74.6)	16,176	(73.7)	11,523	(74.2)	13,590	(74.0)
≥ 25	1,345	(9.3)	2,278	(10.4)	1,452	(9.4)	1,705	(9.3)
**Parity**								
Primipara	5,279	(36.6)	8,197	(37.3)	7,007	(45.1)	9,940	(54.1)
Multipara	8,820	(61.2)	13,290	(60.5)	8,115	(52.3)	7,842	(42.7)
**Smoking status**								
Never	8,784	(60.9)	12,874	(58.6)	9,504	(61.2)	11,616	(63.2)
Former	5,088	(35.3)	8,106	(36.9)	5,485	(35.3)	6,215	(33.8)
Current	465	(3.2)	832	(3.8)	451	(2.9)	434	(2.4)
**Alcohol consumption status**								
Former (stopped before pregnancy)	13,152	(91.2)	19,918	(90.7)	14,266	(91.9)	16,991	(92.5)
Former (stopped after learning of pregnancy)	587	(4.1)	976	(4.4)	657	(4.2)	778	(4.2)
Current	599	(4.2)	934	(4.3)	523	(3.4)	519	(2.8)
**Physical activity during pregnancy, METs・h/day**								
≤ 0.152	3,403	(23.6)	5,187	(23.6)	3,521	(22.7)	4,091	(22.3)
0.157–≤ 1.177	3,492	(24.2)	5,075	(23.1)	3,827	(24.7)	4,469	(24.3)
1.178–≤ 3.305	3,644	(25.3)	5,213	(23.7)	3,883	(25.0)	4,862	(26.5)
≥ 3.310	3,255	(22.6)	5,446	(24.8)	3,572	(23.0)	4,117	(22.4)
**Quintile of folic acid intake during pregnancy, μg**								
< 165	3,946	(27.4)	5,828	(26.5)	3,446	(22.2)	3,763	(20.5)
166–≤ 228	3,637	(25.2)	5,580	(25.4)	3,895	(25.1)	4,459	(24.3)
229–≤ 312	3,543	(24.6)	5,506	(25.1)	4,129	(26.6)	4,834	(26.3)
≥ 312	3,297	(22.9)	5,039	(23.0)	4,054	(26.1)	5,320	(29.0)
**Marital status**								
Married	13,960	(96.8)	21,260	(96.8)	15,098	(97.3)	17,900	(97.4)
Single	244	(1.7)	365	(1.7)	236	(1.5)	266	(1.4)
**Highest education level**								
Junior high school or high school	4,810	(33.3)	7,995	(36.4)	5,009	(32.3)	5,409	(29.4)
Vocational school or junior college	6,013	(41.7)	9,266	(42.2)	6,854	(44.2)	8,113	(44.1)
University or graduate school	3,542	(24.6)	4,591	(20.9)	3,592	(23.1)	4,770	(26.0)
**Highest education level of mother’s partner**								
Junior high school or high school	5,929	(41.1)	9,630	(43.9)	6,377	(41.1)	6,838	(37.2)
Vocational school or junior college	3,225	(22.4)	5,012	(22.8)	3,610	(23.3)	4,212	(22.9)
University or graduate school	5,136	(35.6)	7,085	(32.3)	5,388	(34.7)	7,160	(39.0)
**Employment status**								
Not employed	7,795	(54.0)	11,003	(50.1)	8,011	(51.6)	9,790	(53.3)
Employed	6,443	(44.7)	10,620	(48.4)	7,320	(47.2)	8,358	(45.5)
**Annual household income, million yen**								
< 4	5,486	(38.0)	8,474	(38.6)	5,474	(35.3)	5,850	(31.8)
4–< 6	4,527	(31.4)	6,867	(31.3)	4,978	(32.1)	5,808	(31.6)
≥ 6	3,506	(24.3)	5,160	(23.5)	4,129	(26.6)	5,563	(30.3)
**Use of antibacterial medication during pregnancy**								
No	12,782	(88.6)	19,484	(88.8)	13,755	(88.6)	16,363	(89.0)
Yes	1,554	(10.8)	2,339	(10.7)	1,694	(10.9)	1,914	(10.4)
**Birth weight, g**								
< 2,500	13,242	(91.8)	20,230	(92.2)	14,253	(91.8)	16,821	(91.5)
≥ 2,500	1,153	(8.0)	1,663	(7.6)	1,243	(8.0)	1,508	(8.2)
**Gestational weeks**								
< 37	669	(4.6)	963	(4.4)	664	(4.3)	812	(4.4)
≥ 37	13,733	(95.2)	20,946	(95.4)	14,839	(95.6)	17,524	(95.4)
**Child’s sex**								
Boy	7,517	(52.1)	11,059	(50.4)	7,880	(50.8)	9,583	(52.1)
Girl	6,906	(47.9)	10,894	(49.6)	7,644	(49.2)	8,793	(47.9)
**Cesarean delivery**								
No	11,631	(80.6)	17,973	(81.9)	12,645	(81.5)	14,749	(80.3)
Yes	2,732	(18.9)	3,894	(17.7)	2,817	(18.1)	3,541	(19.3)
**Congenital anomaly**								
No	14,104	(97.8)	21,481	(97.8)	15,189	(97.8)	17,941	(97.6)
Yes	319	(2.2)	472	(2.2)	335	(2.2)	435	(2.4)
**Breastfed at age 1 year**								
No	4,879	(33.8)	8,449	(38.5)	5,919	(38.1)	7,546	(41.1)
Yes	9,488	(65.8)	13,363	(60.9)	9,520	(61.3)	10,720	(58.3)

Using the results of the group that did not consume yogurt as a reference, multivariate logistic regression analysis was performed to compare the neurodevelopment of children according to consumption frequency. The odds ratios for the relationship between frequency of yogurt consumption and neurodevelopment at the age of 3 years are shown in Table 2.

**Table 2:** Odds ratios and 95% confidence intervals for developmental delay in each ASQ-3 domain according to the frequency of yogurt consumption at age 1 year.

ASQ-3	Odds ratio	Frequency of yoghurt intake at age 1 year (times/week)			
		0 (none)	1–2	3–4	≥ 5
Communication skills	Crude	1.00 (Ref)	**0.78(0.70–0.87)**	**0.70(0.62–0.79)**	**0.90(0.81–0.999)**
	Adjusted^a^	1.00 (Ref)	**0.79(0.71–0.88)**	**0.71(0.63–0.80)**	**0.88(0.79–0.98)**
Gross motor skills	Crude	1.00 (Ref)	**0.80(0.72–0.88)**	**0.81(0.73–0.91)**	0.97(0.88–1.10)
	Adjusted^a^	1.00 (Ref)	**0.82(0.74–0.90)**	**0.81(0.72–0.90)**	**0.90(0.81–0.99)**
Fine motor skills	Crude	1.00 (Ref)	**0.87(0.80–0.94)**	**0.83(0.76–0.91)**	1.02(0.94–1.10)
	Adjusted^a^	1.00 (Ref)	**0.87(0.80–0.94)**	**0.82(0.75–0.90)**	0.96(0.88–1.00)
Problem-solving skills	Crude	1.00 (Ref)	**0.81(0.75–0.87)**	**0.78(0.72–0.85)**	**0.87(0.80–0.94)**
	Adjusted^a^	1.00 (Ref)	**0.81(0.75–0.88)**	**0.78(0.72–0.85)**	**0.84(0.77–0.91)**
Personal-social skills	Crude	1.00 (Ref)	**0.77(0.69–0.86)**	**0.73(0.64–0.83)**	0.97(0.86–1.10)
	Adjusted^a^	1.00 (Ref)	**0.78(0.70–0.88)**	**0.73(0.64–0.83)**	0.89(0.80–1.00)

^a^Adjusted for maternal age, body mass index, parity, smoking status, alcohol consumption status, physical activity during pregnancy, folic acid intake during pregnancy, marital status, highest educational level, annual household income, employment status, use of antibacterial medication during pregnancy, birth weight, gestational weeks, child’s sex, caesarean delivery, congenital anomaly, and being breastfeding at age 1 year. **Bold** indicates significance (p < 0.05).

In the group that consumed yogurt 1–4 times/week, the risk of developmental delay was reduced in all ASQ3 domains. However, the group that consumed yogurt ≥ 5 times/week did not have a reduced risk of developmental delay fine motor skills, or personal-social skills.

## Discussion

In this study, we examined the hypothesis that yogurt consumption by children at 1 year of age would be associated with their later neurodevelopmental status at 3 years of age. The results showed that children who consumed yogurt 1–4 times/week had a lower risk of developmental delay at age 3 years compared with children who did not consume yogurt, whereas children who consumed yogurt ≥ 5 times /week showed no decreased risk of developmental delay in several of the ASQ-3 domains used in this evaluation. Taken together, although yogurt consumption at age1 year appears to be associated with a beneficial effect on children’s neurodevelopment, more frequent consumption does not seem to be associated with a greater benefit.

Similar nonlinear results were reported in a study that examined yogurt consumption and the prevalence of common allergic diseases. In that study, yogurt consumption was divided into quartiles (none, small amount, moderate amount, large amount) and the relationship with the prevalence of allergic diseases was analyzed. The results showed a lower prevalence of asthma in the group that consumed a moderate amount of yogurt compared with the group that did not consume any, while a higher prevalence was seen in the group that consumed a large amount [18]. Although many studies have reported that probiotic intake reduced allergic sensitization and allergy morbidity [19–24], there are also reports that probiotic intake promoted allergen sensitization [25], suggesting that there may be an appropriate intake level for probiotics to realize a beneficial effect on the host.

A study conducted in the UK that examined the relationships between yogurt consumption, diet quality, and metabolic diseases in children reported differences in total energy, carbohydrate, and various trace element levels in children who consumed yogurt compared with those who did not, as well as differences in the dietary patterns themselves. The group that consumed more yogurt showed higher dietary quality and higher total energy content. Higher dietary quality is presumed to have beneficial effects on health, and no apparent adverse events were reported in the high consumption group, but there may be negative associations with excessive yogurt consumption that are not yet apparent [26].

Previous studies have shown a relationship between the composition of the gut microbiota and neurodevelopmental disorders [2–5]. The high frequency of gastrointestinal symptoms in children with neurodevelopmental disorders has led to a focus on the relationship between neurodevelopmental disorders and gut bacteria [27–29]. Indeed, a study comparing the gut bacterial composition of children with ASD and those with typical development reported higher proportions of *Clostridium* and *Bacteroides* and lower proportions of Bifidobacterium in children with ASD [30–32]. Habitual consumption of yogurt is known to improve the gut microbiota [6], suggesting that yogurt consumption may be associated with neurodevelopment through the mechanism of improved gut microbiota. Two mechanisms have been postulated for the relationship between gut microbiota and neurodevelopment. The first involves neurotransmitters produced by the gut bacteria themselves. Some gut bacteria are known to produce emotion-related bioactive substances such as GABA, which are speculated to influence cognition and emotion [7, 8].The second mechanism is associated with inflammation of the intestinal tract due to disturbances in the gut microbiota, known as gut dysbiosis. Inflammation of the intestinal tract reduces the local barrier function of the intestinal tract, which in turn leads to the uptake of substances harmful to the human body, substances that can cross the blood–brain barrier and reach the central nervous system, with potentially adverse effects. The presence of inflammation itself is also thought to affect the central nervous system through the production of various cytokines.

Indeed, high levels of inflammatory cytokines in the blood have been reported in several neurodevelopmental disorders and depression [9, 10]. Oral intake of probiotics is expected to prevent the growth of harmful bacteria in the intestinal tract and improve intestinal inflammation [11, 12].

As mentioned above, there are differences in the composition of the gut microbiota between children with neurodevelopmental disorders and those with typical development, but there are two schools of thought as to why this difference arises. One is that neurodevelopmental disorders cause changes in the gut microbiota, and the second is that changes in the gut microbiota cause the symptoms of neurodevelopmental disorders. Santocci et al. investigated the effects of probiotics in children with ASD by assessing the presence of abdominal symptoms such as diarrhea and abdominal pain in addition to the core symptoms of ASD. The results showed that probiotics improved neurodevelopmental symptoms both with and without abdominal symptoms [33]. This means that probiotics can impact neurodevelopment without improving abdominal symptoms. In children with neurodevelopmental disorders, it is likely that the intestinal microbiota is affected by picky eating and other preoccupations. Nevertheless, it may be useful to make efforts to improve the intestinal environment.

Meanwhile, some reports have suggested that there is limited evidence of the effects of probiotics on gastrointestinal symptoms and behavioral disorders in people with ASD. There are no standardized regimens for the type and number of probiotics, making it difficult to demonstrate a clear effect. Although intervention studies for ASD have been conducted, randomized controlled trials have not. Large-scale trials with standardized methods are desired in the future [34].

For foods containing probiotics to be effective, it considered desirable to consume them on a regular basis. This is because orally ingested lactic acid bacteria must adapt to the host’s intestinal environment and be present in the intestinal tract for an extended period to have an effect on the host [35–37].Guerin-Danan et al. studied the effect of yogurt consumption on the intestinal microbiota of healthy infants aged 10–18 months and found that consumption was associated with an increased the number of lactobacilli in feces [38]. Another study reported the detection of lactobacilli in feces for up to 8 days after consumption was discontinued [39], suggesting that these bacteria can survive in the intestinal tract for at least that long. However, it is also known that the bacteria in yogurt do not settle in the intestinal tract [40], and habitual intake is necessary to obtain a lasting effect. The formation of the intestinal microbiota begins immediately after birth, and the prototype for an individual’s intestinal microbiota is formed in the first week of life [41]. Therefore, regular intake of probiotics early in infancy may be beneficial for improving the intestinal environment as it is modified by the food ingested and other external factors. Because the intestinal environment also affects the nervous system, regular intake of probiotics and probiotic-containing foods in developing children may also have a positive association with later neurodevelopment.

The strengths of this study include the large sample size of over 70,000 mother–child pairs from across Japan and the sample being collected within the past 10 years. Accordingly, the sample is considered representative of recent Japanese mother–child pairs. There have been no other large-scale studies investigating the association between dietary intake in early childhood and later neurodevelopment to our knowledge, making this the first report on the association between yogurt consumption and subsequent neurodevelopmental status in early childhood.

The novelty of this study is that it assessed the relationship between dietary intake at younger ages and later development, and showed just such an association. Previous studies have examined the effects of administering probiotics to children with a diagnosis of some form of neurodevelopmental disorder, but the present study confirms the effects of intervention at a younger age. In addition, the intervention was set up as a food (yogurt), which can be consumed on a daily basis, rather than a drug such as a probiotic preparation, making it easier to incorporate into daily life.

This study also has some limitations that must be considered. First, the ASQ-3 was used to assess neurodevelopment and although the ASQ-3 is a well-validated questionnaire, it is neither diagnostic nor objective. Second, the types of yogurt consumed by the participating children were not reported and thus the details of the probiotics they actually consumed are not known. Third, because we did not check the intestinal bacteria of the children, it is not clear whether there was any actual change in the intestinal environment. Fourth, in this study, participants born to the same mother were excluded to avoid the possibility of introducing genetic or environmental bias in the results. We also excluded participants with unknown yogurt intake status, which was set as the exposure for this study. Consequently, a large number of participants were excluded, which may have affected the generalizability of the results. Fifth, as mentioned above, it has been speculated that there is an association between yogurt intake and diet quality [26], and it is possible that the association found here is influenced by dietary content other than yogurt. Finally, this study evaluated only the frequency of yogurt consumption and not the quantity, so further studies focusing on both frequency and quantity are needed.

In conclusion, we found that regular intake of yogurt at age 1 year was associated with a lower risk of developmental delay at the age of 3 years compared with no consumption. However, more frequent consumption did not necessarily mean a lower risk. Further studies on this association, including intervention studies, are warranted.
